# Yi Guan Jian, a Traditional Chinese Herbal Medicine, Alleviates Carbon Tetrachloride-Induced Liver Injury

**DOI:** 10.1155/2019/9824728

**Published:** 2019-02-12

**Authors:** Xu Lixin, Gu Erli, Huang Songping, Zhu Yonggen, Jianping Wang, Yan Lijun

**Affiliations:** ^1^Nantong Third People's Hospital, Nantong University, Nantong, China; ^2^Outpatient Department, Affiliated Hospital of Nantong University, Nantong, China

## Abstract

**Objective:**

To study the protective effect of Yi Guan Jian (YGJ) on liver injury induced by carbon tetrachloride (CCl_4_) in mice.

**Methods:**

50 ICR mice were randomly divided into 5 groups: control group, carbon tetrachloride (CCl_4_) group, CCl_4_ + silymarin Group (200 mg/kg), carbon CCl_4_ + YGJ Group (11.5, 23 g/kg). Except the mice in the control group and the CCl4 group that were given the same volume of distilled water, the mice in other groups were given the drugs for seven days. 2 hours after the last administration, except the mice in the control group, the mice in other groups had intraperitoneal injection of 0.1% CCl_4_ vegetable oil solution (10 ml/kg). Mice in control group had intraperitoneal injection of the same volume of vegetable oil. After 18h, the blood and liver were collected. The liver of mice was stained with HE staining, the levels of alanine transaminase (ALT) and glutamic pyruvic transaminas (AST) in serum were detected, malondialdehyde (MDA), superoxide dismutase (SOD), interleukin (IL-6, Il-1*β*), and tumor necrosis factor (TNF-*α*) in serum and liver were detected, and the Western blot was used to detect the levels of MAPK/NF-*κ*B pathway.

**Results:**

YGJ significantly decreased the levels of ALT and AST. The contents of MDA, IL-6, Il-1*β*, and TNF-*α* in serum and liver were significantly decreased and the contents of SOD in serum and liver were significantly decreased by YGJ, which significantly improved the pathological changes of liver tissue in mice. The levels of MAPK/NF-*κ*B pathway were significantly decreased by YGJ.

**Conclusion:**

YGJ has protective effect on CCl_4_ induced liver injury in mice, which may be related to the MAPK/NF-*κ*B signaling pathway.

## 1. Introduction

The liver is one of the most important substantive organs of human body. It has strong metabolic and defensive functions. When various foreign chemicals or wastes generated by metabolism accumulate in the liver, liver injury, hepatitis, hepatic fibrosis, and even hepatocyte necrosis may occur. These chemical substances include carbon tetrachloride (CCl_4_), alcohol, paracetamol, and other cold medicines [1]. Therefore, liver disease has become one of the common diseases affecting human health. At present, the treatment of liver diseases mainly focuses on stopping taking drugs that may cause liver injury immediately and treating them with anti-inflammatory, anti-free radical damage, protecting hepatocyte membrane, promoting hepatocyte metabolism, promoting hepatocyte repair and regeneration, and promoting bilirubin and bile acid metabolism [2]. Traditional Chinese medicine is rich in resources, and many Chinese herbal medicines have certain protective effects on liver. The use of traditional Chinese medicine to treat liver injury has gradually attracted the attention of scholars at home and abroad.

The animal models of chemical liver injury include carbon tetrachloride, d-galactosamine, and acetaminophen, among which carbon tetrachloride is the most classic acute liver injury model, with good repeatability and simple technology. Carbon tetrachloride is a cytotoxic substance that can be rapidly absorbed by the liver. It can induce free radical formation in the liver and lead to chain peroxidation [3]. At the same time, carbon tetrachloride can also cause the disorder of cellular environment and activate a large number of inflammatory factors, thus further producing oxygen free radicals and aggravating liver injury. Therefore, carbon tetrachloride was used in this experiment to replicate the liver injury model of mice.

Liver disease caused by alcohol, viral infection, or nonalcoholic steatohepatitis causing severe hepatocyte injury is highly associated with acute or chronic inflammation [4, 5]. In the process of liver inflammation, a large number of types of cells, such as natural killer cells, T cells, dendritic cells, and macrophages, are absorbed. Macrophages in the liver play a key role in stimulating liver injury because inflammatory cytokines (including TNF-*α*, IL-1*β*, and IL-6) and reactive oxygen species are produced in large quantities under inflammatory stimulus [6]. Administration of carbon tetrachloride (carbon tetrachloride) to mice is a classic experimental model of severe liver injury, involving the production of inflammatory cytokines and the supplementation of inflammatory cells, resulting in liver structural damage and dysfunction [7].

Silymarin is a promising drug for the treatment of acute liver injury induced by carbon tetrachloride. Silymarin is a herbal medicine for protecting liver and contains four flavonoid lignans isomers, including 60-70 % silybin, 20 % silybin, 10 % silybin, and 5 % isosilybin. Silymarin has been well demonstrated to exert multiple beneficial effects and thus used as a natural remedy for the treatment of hepatitis, jaundice, and cirrhosis [8]. It protects against liver injury induced by radiation, alcohol abuse, ischemia, iron overload, environmental toxins, and CCl_4_. Many scholars used silymarin as a positive drug to study the liver protection effect of other drugs [9].

At present, the prevention and treatment of liver injury are very common in both western medicine and traditional Chinese medicine. The new guidelines show that health education and lifestyle intervention are still the first choice for treatment of liver injury [10]. According to this treatment method, the prevention and treatment of liver injury and liver injury by single herbal medicine, compound Chinese medicine, and their effective components have become a hot topic in this field. Yi Guan Jian as the main drug for treating liver depression symptoms is recorded in the classic book “*Xu Ming Yi Lie an Xin Wei Tong Men.*” Modern pharmacology reveals that Yi Guan Jian has hepatoprotective and anti-inflammatory effects, but its mechanism is not well explained. Therefore, our study investigated the effects of Yi Guan Jian on CCl4-induced liver injury.

## 2. Materials and Methods 

### 2.1. Yi Guan Jian Preparation

All the herbs of Yi Guan Jian were purchased from Jiangyin Tianjiang Pharmaceutical Co., Ltd. (Jiangsu, China). The aqueous extract of Yi Guan Jian was prepared according to the following procedures: these herbs were soaked in 6 times (v/w) distilled water for an hour and then heated to boiling and decocted for 30 min. The filtrate was then collected. The residue was decocted for 20 min with 4 times (v/w) distilled water, and then the filtrate was collected and mixed with the previously collected filtrate and stored at 4°C until use.

### 2.2. HPLC Analysis of Yi Guan Jian

HPLC was performed using Agilent 1200 HPLC with G1321A FD, Eclipse AAA column (4.6×150 mm, 5 *μ*m), and a column temperature of 40°C. Mobile phase A (formic acid: water =1:1000) and mobile phase B (acetonitrile). The gradient elution procedure was 0–13 minutes, B (0%–63%), 0–5 minutes, B (63%), 5-8 minutes, B (100%), 9 minutes, B (100%–0%), and 10-13 minutes, B (0%). The flow rate was 0.4 mL min^−1^.

### 2.3. Animals

Fifty male ICR mice (20-22 g ) were provided by the experimental animal center of Nantong university. Certificate No.: scxk (su) 2012 - 0001. They were housed in an air-conditioned room at 23 ± 2°C with a 12-h light/dark cycle. Water and food were provided ad libitum. All the experimental procedures were performed according to the National Institutes of Health Guidelines for the Care and Use of Laboratory Animals.

### 2.4. Reagents

The commercial AST, ALT, MDA, and SOD kits were purchased by Jiancheng Bioengineering Institute (Nanjing, China). Enzyme-linked immunosorbent assay (ELISA) tests for the detection of IL-6, IL-1*β*, and TNF-*α* were produced by Nanjing KeyGEN Biotech. Co., Ltd. (Nanjing, China). All the antibodies were provided by Cell Signaling Technology (Danvers, USA).

### 2.5. Experimental Protocol

50 ICR mice were randomly divided into 5 groups: control group, carbon tetrachloride (CCl_4_) group, CCl_4_ + silymarin Group (positive drug, 200 mg/kg), and carbon CCl_4_ + YGJ Group (11.5, 23 g/kg). Except the mice in the control group and the CCl4 group that were given the same volume of distilled water, the mice in other groups were given the drugs for seven days. 2 hours after the last administration, except the mice in the control group, the mice in other groups had intraperitoneal injection of 0.1% CCl_4_ vegetable oil solution (10 ml/kg). Mice in control group had intraperitoneal injection of same volume of vegetable oil. After 18h, the blood and liver were collected.

### 2.6. Histopathological Observation of Liver

After the mice were killed by taking blood from the orbit, the liver tissue was fixed in 4 % neutral formalin solution. The tissue was embedded in paraffin wax and cut into 4 mm thick slices. Paraffin was removed and stained with hematoxylin-eosin dye (HE stain). The histopathological changes of liver were observed by optical microscope.

### 2.7. Determination of AST, ALT, SOD, and MDA in Serum

Blood was collected from the orbit of mice. After centrifugation at 4500 rpm for 15 min, the supernatant was frozen at-80°C for later use. The contents of AST, ALT, SOD, and MDA in serum of mice were determined according to the instructions of the kits.

### 2.8. Determination of Inflammatory Cytokines in Serum and Liver

The determination of inflammatory cytokines in serum and liver was performed by the methods of Liu et al. [11]. Briefly, mice were anesthetized with 10 % chloral hydrate, blood was collected from orbital veins, centrifuged for 30 min at 3000 r/min, and supernatant was extracted at 4°C for later use. At the same time, liver tissue was taken, weighed, and precooled, PBS buffer was added, homogenized on ice with a glass homogenizer, and centrifuged at a low temperature of 12 000 r/min for 15 min, and supernatant was extracted at 4°C for later use. Serum and tissue supernatants were used to detect the contents of IL-6, IL -1*β*, and TNF-*α*, and the experimental procedures were strictly in accordance with the instructions of ELISA kit. The protein content of liver tissue was detected by BCA method. The contents of I IL-6, IL -1*β*, and TNF-*α* in liver tissue need to be compared with the protein content of liver.

### 2.9. Western Blot

The methods of Western blot for JNK, ERK, P38, P65, and IkBa and phosphorylated JNK, ERK, P38, P65, and IkBa in liver were performed according to the methods of Liu et al. [11]. Briefly, mice in each group were randomly selected, liver tissue was separated on ice, scissors were cut and weighed, lysate was added, homogenized on ice with a glass homogenizer, and centrifuged at a low temperature of 12000 r/min for 10 min, supernatant was extracted, protein was quantified by BCA method, and 5× LAEM MIL protein loading buffer was added and stored in a water bath of 100°C for 5 min and -80°C for later use. After 10 % SDS - PAGE, the gel was concentrated at 80 V for 15 min and the gel was separated at 120 V until bromophenol blue ran to the bottom of the gel. After 1 h of film transfer, 5 % skimmed milk powder was sealed at 4°C overnight, TBST was washed, I was added to resist room temperature for 1 h, TBST was washed, horseradish peroxidase labeled II was added to resist room temperature for 1 h, TBST was washed, the film was placed in a gel imager, ECL light-emitting agent was added, exposure and visualization were performed, and protein levels were analyzed using Image J software

### 2.10. Statistical Analysis

The statistical processing was analyzed by SPSS 13. 0 statistical software. The data were expressed by means ± SDs and two independent samples t test were used for comparison, with p < 0.05 as the difference being statistically significant.

## 3. Results

### 3.1. HPLC Analysis of Yi Guan Jian

**Figure 1:**
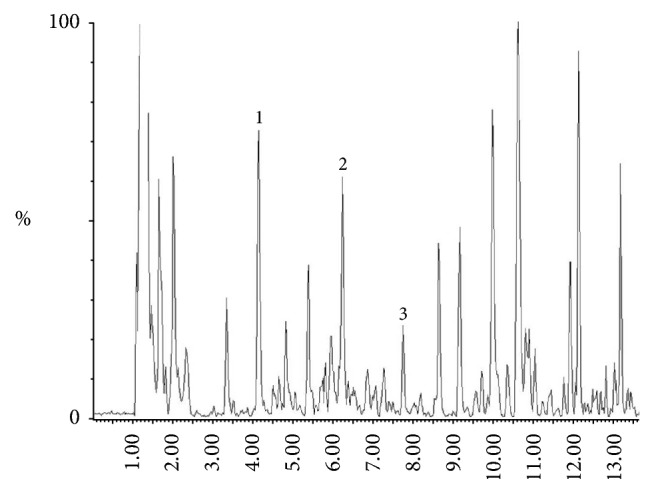
HPLC analysis of Yi Guan Jian. 1: ginsenoside Rg1. 2: leonuride. 3: aucubin. 4: verbascoside. 5: ferulic acid.

As data show in the Figure 1, three compounds ginsenoside Rg1, leonuride, and aucubin were identified, and the contents of ginsenoside Rg1, leonuride, aucubin, verbascoside, and ferulic acid were 0.107 ug/mg and 0.542 ug/mg, 0.319 ug/mg, 0.252ug/kg, and 0.491ug/mg.

### 3.2. Effects of Yi Guan Jian on MDA and SOD in Serum of Mice

**Figure 2:**
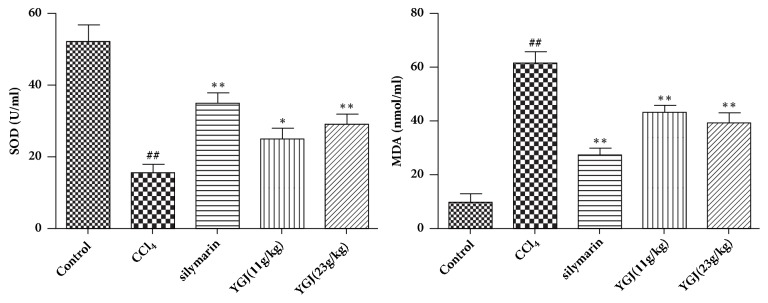
Effects of Yi Guan Jian on MDA and SOD in serum of mice. The data are expressed as mean values ± SDs. ^#^p < 0.05 and ^##^p < 0.01 compared with control group. ^*∗*^p < 0.05 and ^*∗∗*^p < 0.01 compared with CCl_4_ group.

As shown in Figure 2, compared with the control group, the MDA content increased significantly and the activity of SOD decreased significantly in the model group mice after injecting CCl_4_, and compared with the model group, Yi Guan Jian and silymarin can significantly reduce MDA content and enhance SOD activity. It is indicated that Yi Guan Jian may improve CCl4 induced liver injury by alleviating oxidative stress.

### 3.3. Effects of Yi Guan Jian on AST and ALT in Serum of Mice

**Figure 3:**
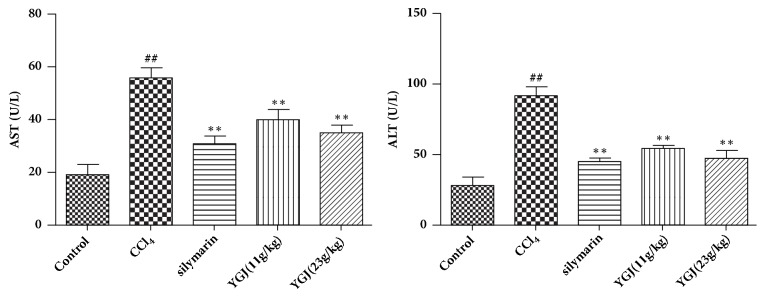
Effects of Yi Guan Jian on AST and ALT in serum of mice. The data are expressed as mean values ± SDs. ^#^p < 0.05 and ^##^p < 0.01 compared with control group. ^*∗*^p < 0.05 and ^*∗∗*^p < 0.01 compared with CCl_4_ group.

As shown in Figure 3, compared with the control group, the AST and ALT content increased significantly in the model group mice after injecting CCl4, and compared with the model group, Yi Guan Jian and silymarin can significantly reduce M AST and ALT content.

### 3.4. Effects of Yi Guan Jian on TNF-*α*, IL-6, and IL-1*β* in Serum and Liver

**Figure 4:**
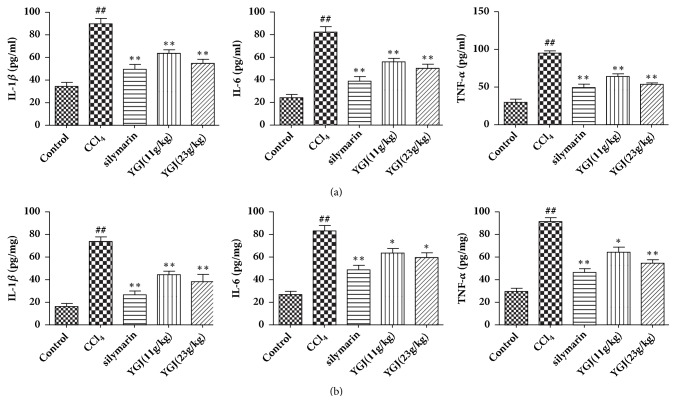
Effects of Yi Guan Jian on TNF-*α*, IL-6 and IL-1*β* in serum (a) and liver (b). The data are expressed as mean values ± SDs. ^#^p < 0.05 and ^##^p < 0.01 compared with control group. ^*∗*^p < 0.05 and ^*∗∗*^p < 0.01 compared with CCl_4_ group.

Inflammatory cytokines are one of the main indicators of liver injury. The levels of TNF-*α*, IL-6, and IL-1*β* in serum and liver were evaluated in CCl_4_ mice. Compared with rats in CCl_4_ group, the serum and liver TNF-*α*, IL-6, and IL-1*β* levels in Yi Guan Jian treatment group were significantly lower, respectively (Figure 4).

### 3.5. Effects of Yi Guan Jian on Pathological Changes

**Figure 5:**
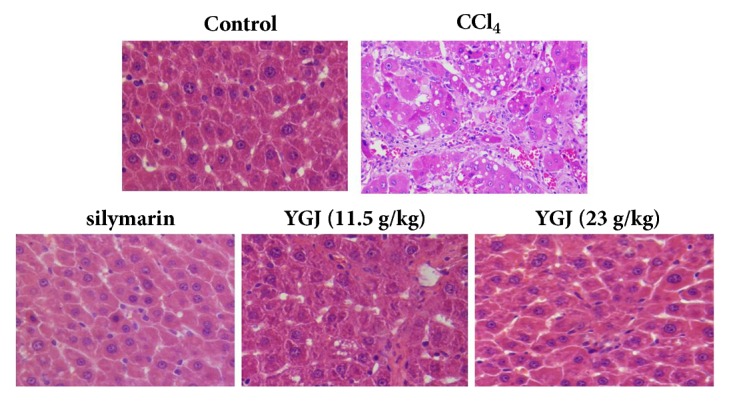
Effects of Yi Guan Jian on pathological changes (x400).

Histopathological examination results are shown in Figure 5, the structure of liver tissue in the blank control group was normal, the structure of the hepatic lobules was clear, the cell line was arranged, and the cells were in normal shape. In CCl_4_ group mice, the hepatic lobules were blurred, the hepatic cord was disordered, and the hepatocyte degeneration and inflammatory cells infiltration were obvious. Compared with the model group, the structure of hepatic lobules in Yi guan Jian and silymarin was basically restored to normal, the hepatocyte morphology was normal, and inflammatory cell infiltration decreased.

### 3.6. Effects of Yi Guan Jian on MAPK/NF-*κ*B Pathway in Liver

**Figure 6:**
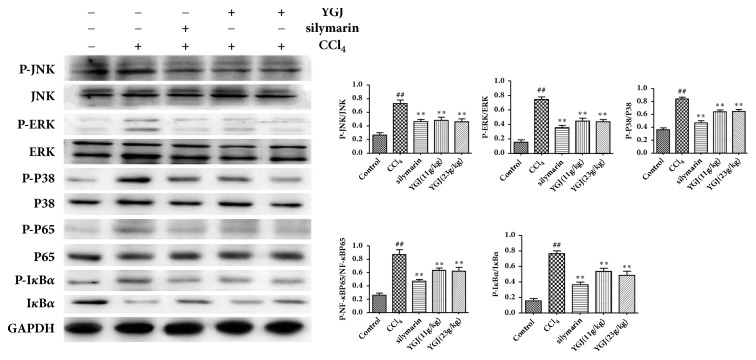
Effects of Yi Guan Jian on MAPK/NF-*κ*B pathway in liver. The data are expressed as mean values ± SDs. ^#^p < 0.05 and ^##^p < 0.01 compared with control group. ^*∗*^p < 0.05 and ^*∗∗*^p < 0.01 compared with CCl_4_ group.

Western blot was used to detect the related protein expression of Yi Guan Jian in CCl4-induced liver injury mice, and its related mechanism has been determined. As shown in Figure 6, compared with the control group, the expression of MAPK/NF-*κ*B pathway was upregulated. Compared with the model group, the expression of MAPK/NF-*κ*B pathway in liver tissue of mice in Yi Guan Jian and silymarin groups decreased. The results showed that Yi Guan Jian improves liver injury induced by CCl_4_ via MAPK/NF-*κ*B pathway.

## 4. Discussion

The liver is the largest metabolic organ in the organism. Many metabolic products and exogenous substances will cause liver injury. Long-term foreign aid stimulation may even cause liver fibrosis, cirrhosis, and other pathological phenomena. It is a disease that seriously endangers human health. The liver injury model of carbon tetrachloride (CCl_4_) is a classic model. This model is commonly used at home and abroad to study the efficacy and mechanism of hepatoprotective drugs. When CCl_4_ enters liver tissue, it generates free radicals under the action of liver microsomal cytochrome P450, which can combine with molecules in liver cells and also act on lipid substances in liver cell membranes, causing lipid peroxidation damage [12]. At the same time, CCl4 produces a large number of free radicals under the action of the liver, which combine with phospholipids in the cell membrane to produce lipid peroxides, and its metabolite malondialdehyde (MDA) can also cause cell damage.

Oxidative stress refers to the imbalance of oxidation and antioxidation in vivo, which results in the infiltration of neutrophils, the increase of protease secretion, and the production of a large number of oxidation intermediate products. Oxidative stress is one of the important factors leading to disease and aging. A large number of studies have shown that stimulation of an exogenous substance can lead to activation of the oxidative stress system [13]. Superoxide dismutase (SOD) is an important antioxidant enzyme in organism, which is the primary substance of scavenging free radicals. The results showed that the content of MDA increased significantly and the activity of SOD significantly decreased in model group, while the Yi Guan Jian and Silymarin could significantly reduce MDA content and enhance SOD activity. It is indicated that Yi Guan Jian may improve CCl_4_ induced liver injury by alleviating lipid peroxidation and oxidative stress.

Inflammatory cytokines are endogenous peptides with powerful biological effects in vivo, which are produced by immune system cells and can mediate multiple immune responses. IL-1*β*, IL-6, and TNF-*α* are common inflammatory cytokines, and this study shows that the levels of inflammatory cytokines in the serum IL-1*β*, IL-6, and TNF-*α* are significantly elevated in the model group CCl_4_. Yi Guan Jian and silymarin can significantly reduce serum and liver levels of IL-1*β*, IL-6, and TNF-*α*. It is indicated that Yi Guan Jian may improve CCl_4_ induced liver injury by relieving inflammatory reaction.

Many exogenous compounds can cause the activation of MAPK/NF-*κ*B signaling pathways. MAPK activation led to activate NF-*κ*B. I*κ*B is NF-*κ*B inhibition protein, in the resting state, I*κ*B*α* and NF-*κ*B exist in the cytoplasm, and when the I*κ*B*α* stimulated by phosphorylation, NF-*κ*B will activate into the nucleus and induce inflammation reaction. The results showed that the expression of p-JNK, P-ERK, p-P38, p-I*κ*B*α*, and p-NF-*κ*Bp65 in the liver tissue was increased after the injection of CCL_4_, while the Yi Guan Jian and silymarin could significantly reduce p-JNK, P-ERK, p-P38, p-I*κ*B*α*, and p-NF-*κ*Bp65 in liver tissue. It is indicated that the Yi Guan Jian may improve the liver injury induced by CCl_4_ in mice via regulation of MAPK/NF-*κ*B signaling pathway.

To sum up, this experiment shows that Yi Guan Jian can improve liver injury induced by CCl_4_ in mice through regulation of MAPK/NF-*κ*B signaling pathway, and the specific mechanism needs further study.

## Data Availability

The data used to support the findings of this study are available from the corresponding author upon request.
